# Efficacy of Gene Therapy Is Dependent on Disease Progression in Dystrophic Mice with Mutations in the FKRP Gene

**DOI:** 10.1016/j.omtm.2017.02.002

**Published:** 2017-03-08

**Authors:** Charles Harvey Vannoy, Will Xiao, Peijuan Lu, Xiao Xiao, Qi Long Lu

**Affiliations:** 1 McColl-Lockwood Laboratory for Muscular Dystrophy Research, Cannon Research Center, Carolinas Medical Center, Carolinas Healthcare System, Charlotte, NC 28203, USA; 2 Division of Molecular Pharmaceutics, Eshelman School of Pharmacy, University of North Carolina at Chapel Hill, Chapel Hill, NC 27599, USA

**Keywords:** adeno-associated virus, dystroglycanopathy, fukutin-related protein, gene therapy, muscular dystrophy

## Abstract

Loss-of-function mutations in the Fukutin-related protein (*FKRP*) gene cause limb-girdle muscular dystrophy type 2I (LGMD2I) and other forms of congenital muscular dystrophy-dystroglycanopathy that are associated with glycosylation defects in the α-dystroglycan (α-DG) protein. Systemic administration of a single dose of recombinant adeno-associated virus serotype 9 (AAV9) vector expressing human *FKRP* to a mouse model of LGMD2I at various stages of disease progression was evaluated. The results demonstrate rescue of functional glycosylation of α-DG and muscle function, along with improvements in muscle structure at all disease stages versus age-matched untreated cohorts. Nevertheless, mice treated in the latter stages of disease progression revealed a decrease in beneficial effects of the treatment. The results provide a proof of concept for future clinical trials in patients with *FKRP*-related muscular dystrophy and demonstrate that AAV-mediated gene therapy can potentially benefit patients at all stages of disease progression, but earlier intervention would be highly preferred.

## Introduction

Dystroglycanopathies are a subgroup of muscular dystrophy that result from aberrant glycosylation of α-dystroglycan (α-DG) and are characterized pathologically by muscle fiber degeneration and clinically by progressive muscle weakness.^1^ These disorders are caused by mutations in a multitude of genes, including the gene encoding Fukutin-related protein (*FKRP*; OMIM 606596). The *FKRP* gene, along with *fukutin*, is part of a family of genes that possess the putative catalytic DXD motif—a conserved motif found in many families of glycosyltransferases—and is predicted to function as a ribitol 5-phosphate transferase, which is fundamentally required for the post-translational modification of α-DG.^2^, ^3^, ^4^, ^5^ In normal tissues, the mucin-like domain of α-DG is modified with numerous oligosaccharides that are essential for function—anchor the structural framework inside each cell (cytoskeleton) to the lattice of extracellular matrix proteins.^6^, ^7^, ^8^ Missense mutations in the *FKRP* gene lead to FKRP protein deficiencies that impair the glycosylation pathway of α-DG.^9^, ^10^ As a consequence of reduced FKRP protein activity, α-DG is hypoglycosylated and has a reduced binding capacity to the extracellular matrix proteins, effectively weakening the bridge between the dystrophin-glycoprotein complex and the extracellular matrix and disrupting the skeletal muscle basal lamina.^11^

Mutations in the *FKRP* gene exhibit a wide spectrum of clinical severity, ranging from severe congenital muscular dystrophies to limb-girdle muscular dystrophy type 2I (LGMD2I).^9^, ^10^, ^12^ LGMD2I can present as mild or severe depending on the age of onset. Early childhood onset of LGMD2I usually indicates a severe clinical course with affected individuals becoming non-ambulatory as early as their teens. The late- or adult-onset form of LGMD2I is a slowly progressive, milder form of the disorder. Respiratory and cardiac involvement is prominent in all disease severities.^13^ The variable phenotypic severity has been partly attributed to the differences in location of point mutations within the coding sequence, affecting protein transportation and its glycosyltransferase activity differentially.^14^, ^15^, ^16^ Therefore, it is likely that any type of therapeutic intervention could potentially have different efficacies for individuals at different stages of disease progression.

Currently, recombinant adeno-associated virus (AAV) gene therapy is one of the most promising strategies for replacing a gene with loss-of-function mutations. AAV is a small (∼25 nm in diameter) human parvovirus that packages a linear single-stranded DNA genome and is replication defective. The lack of pathogenicity of the virus and its ability to persist stably in transduced cells, especially in post-proliferative muscle tissues, make it a desirable and effective delivery vehicle for gene therapy applications to muscular dystrophies.^17^, ^18^ As a result, AAV gene therapy has been tested in various animal models of many limb-girdle muscular dystrophies,^19^, ^20^, ^21^, ^22^, ^23^, ^24^ along with ongoing/completed clinical trials for dysferlinopathy (ClinicalTrials.gov identifier NCT02710500), LGMD2C (γ-sarcoglycan) (ClinicalTrials.gov identifier NCT01344798), and LGMD2D (α-sarcoglycan) (ClinicalTrials.gov identifier NCT00494195).

In this study, an AAV serotype 9 vector containing a full-length human *FKRP* gene (AAV9-*FKRP*) under control of a muscle creatine kinase-based promoter was administered by a single intravenous tail-vein injection at a dose of 2.5 × 10^13^ vector genomes per kilogram (vg/kg) at onset or later stages of disease progression in our dystrophic mouse model containing a missense mutation in the *FKRP* gene. Our objective is to systematically evaluate the therapeutic potential of AAV-mediated *FKRP* gene delivery and assess the efficacy of the gene replacement therapy for individuals with LGMD2I exhibiting various degrees of disease pathology before a viable treatment option is available.

## Results

### Systemic *FKRP* Delivery Restores Functional α-DG in Dystrophic Mice

**Figure 1:**
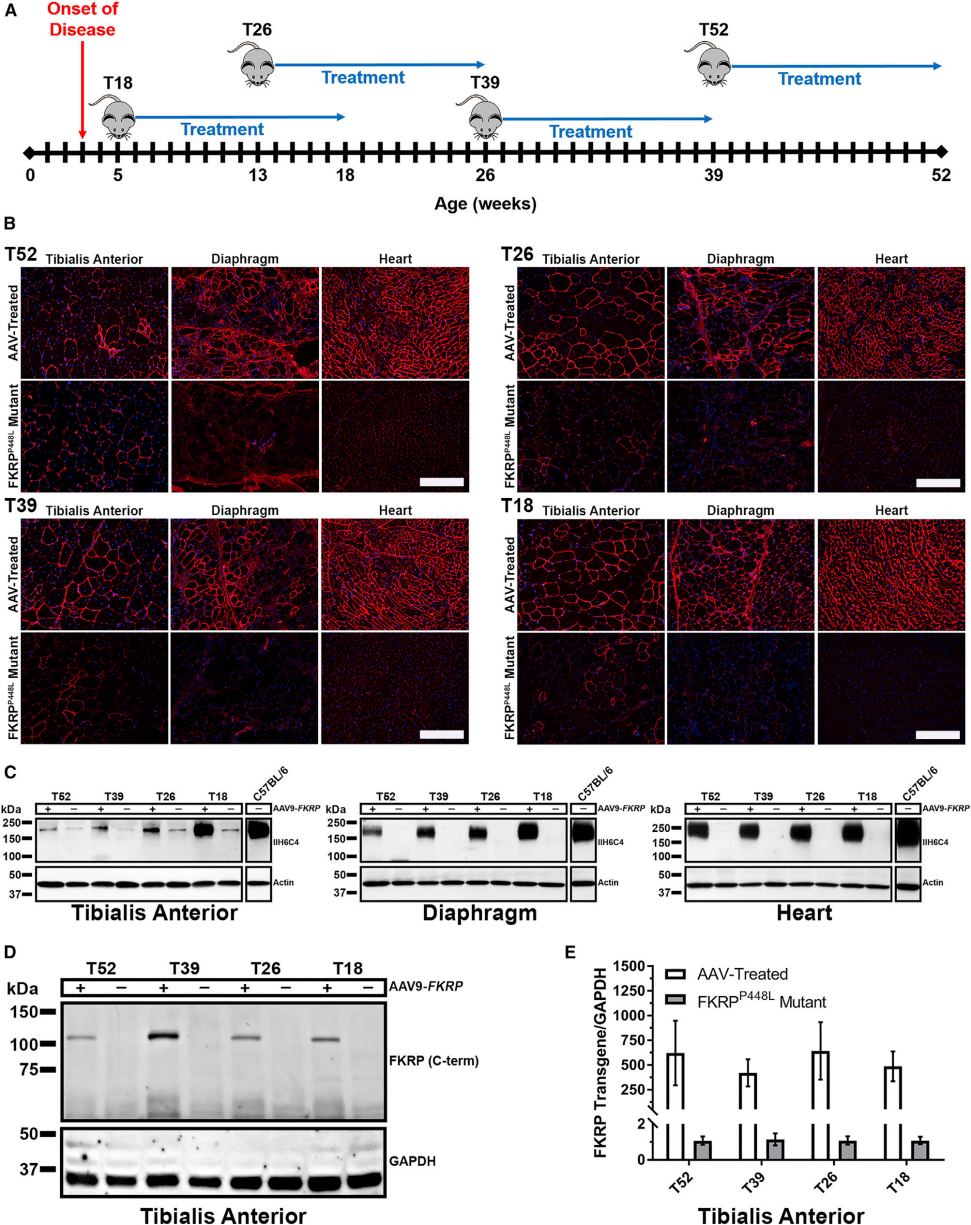
Study Design and Rescue of α-DG Glycosylation in Dystrophic Mice (A) Experimental design of FKRP^P448L^ mutant mice injected with AAV9-*FKRP* at 5 (T18, n = 4), 13 (T26, n = 4), 26 (T39, n = 4), and 39 weeks of age (T52, n = 4) in a 13-week treatment study. (B) Immunofluorescence staining of glycosylated α-DG in tibialis anterior, diaphragm, and heart tissue from indicated mice. Scale bars, 200 μm. (C) Western blot analysis of glycosylated α-DG expression in tibialis anterior, diaphragm, and heart tissue from FKRP^P448L^ mutant mice injected with AAV9-FKRP (+) or untreated (−). Glycosylated α-DG was detected with the IIH6C4 antibody at a dilution of 1:200 and 1:1,000 for immunofluorescence staining and western blot, respectively. Anti-actin antibody was used as the protein loading control. (D) Western blot analysis of exogenous FKRP protein in tibialis anterior tissues from indicated mice. FKRP was detected with an FKRP (C-terminal) antibody at a dilution of 1:400. Anti-GAPDH antibody was used as the protein loading control. (E) FKRP transgene expression in tibialis anterior tissues was analyzed by quantitative real-time PCR methods. All levels are relative to those in untreated FKRP^P448L^ mutant mice cohorts and have been normalized using GAPDH.

For these experiments, we used a mouse model containing a homozygous missense mutation (c.1343C > T, p.Pro448Leu) in the *FKRP* gene (FKRP^P448L^ mutant), as previously described.^25^, ^26^ Onset of the dystrophic attributes can be observed as early as 3 weeks, with progressive pathological changes as the mouse ages. Accordingly, we formulated four age groups of FKRP^P448L^ mutant mice (5, 13, 26, and 39 weeks of age) representing disease progression from an early stage, when muscle degeneration has just become clearly identifiable, to a late stage characterized by severe dystrophy and fibrosis in skeletal muscles and noticeable defects in cardiac muscle function. To correct for the *FKRP* deficiency, we utilized a skeletal/cardiac muscle-tropic AAV serotype 9 vector expressing a full-length human *FKRP* coding sequence under control of a muscle-specific creatine kinase-based (CK7) promoter (AAV9-CK7-Hu*FKRP*, abbreviated as AAV9-*FKRP*) (Figure S1). A single tail-vein injection of AAV9-*FKRP* at a dose of 2.5 × 10^13^ vg/kg was administered to all age groups. Mice were monitored and functionally assessed for a 13-week period and then were subsequently euthanized for analysis, giving rise to cohorts T18, T26, T39, and T52, respectively (Figure 1A). Age-matched untreated FKRP^P448L^ mutant mice were used as negative controls. All mice remained healthy in appearance, activity, and body weight over the 13-week observation period.

Analysis of glycosylation on α-DG by immunohistochemistry at euthanasia demonstrates that intravenous delivery of AAV9-*FKRP* reconstituted functional glycosylation of α-DG in skeletal muscles throughout the body, including the heart (Figure 1B). Age-dependent variation in levels of functional glycosylation of α-DG was visualized by immunohistochemistry with a monoclonal anti-α-DG antibody (IIH6C4) specific to glycosylated epitopes on α-DG,^27^ also known as functional α-DG. The IIH6C4 signals were localized to the sarcolemma and relatively homogeneous in a large portion of muscle fibers and were generally higher in the diaphragm and heart compared to the skeletal muscles, which is likely due to the higher tropism of AAV9 to the cardiac muscle. Positive signals were relatively stronger in the muscles of the early-stage treatment cohorts in comparison to the late-stage cohorts, especially in the skeletal muscles. In stark contrast, a positive immunofluorescence signal for functionally glycosylated α-DG was undetectable in all muscles of the age-matched untreated cohorts, except for a few revertant fibers in the tibialis anterior muscles. Western blot analysis confirmed that the AAV-treated muscle produced normal glycosylated forms of α-DG (150–250 kDa; carbohydrate composition differs depending on tissue type) similar to that detected in the same muscle type of wild-type tissue (Figure 1C). Similar to the immunofluorescence staining, the levels of functional glycosylation were evidently different in all tissues between the early- and late-stage treatment cohorts, with the highest levels in the T18 cohort and lowest levels in the T52 cohort. Only 12%, 34%, and 53% of normal levels of functionally glycosylated α-DG were detected in the tibialis anterior, diaphragm, and heart muscles of the oldest T52 age cohort, respectively, whereas approximately 87%, 88%, and 70% of normal levels were detected in the corresponding three muscles from the youngest T18 cohort. Interestingly, an age-related decrease in levels of functionally glycosylated α-DG was also muscle-type dependent, predominantly in the tibialis anterior muscle. As expected, there was little or no immunoreactivity detectable in the age-matched untreated FKRP^P448L^ mutant samples. IIH6C4 expression in the tibialis anterior was representative of all skeletal muscles tested (quadriceps, gastrocnemius, bicep, and triceps; data not shown).

Western blot (Figure 1D) and quantitative real-time PCR analysis (Figure 1E) demonstrated similar levels of FKRP transgene expression in the AAV-treated cohorts. Expression of vector-driven FKRP protein was analyzed on whole-muscle lysates by western blotting with an antibody raised against a peptide mapping near the C terminus of FKRP (human origin). FKRP protein was clearly detected in the tibialis anterior muscle of all AAV-treated cohorts, with a distinct signal band at approximately 110 kDa that corresponds to a dimeric FKRP. The intensity of the FKRP signal was similar for all AAV-treated cohorts, whereas it was undetectable in all age-matched untreated FKRP^P448L^ mutant samples. We also validated the expression level of the FKRP transgene by quantitative real-time PCR analysis in all AAV-treated and age-matched untreated FKRP^P448L^ mutant cohorts. These results show that a single injection of AAV9-*FKRP* is efficacious in rescuing functional glycosylation of α-DG in FKRP^P448L^ mutant mice at all stages of disease pathology.

### AAV9-*FKRP* Gene Therapy Improves Muscle Pathology

**Figure 2:**
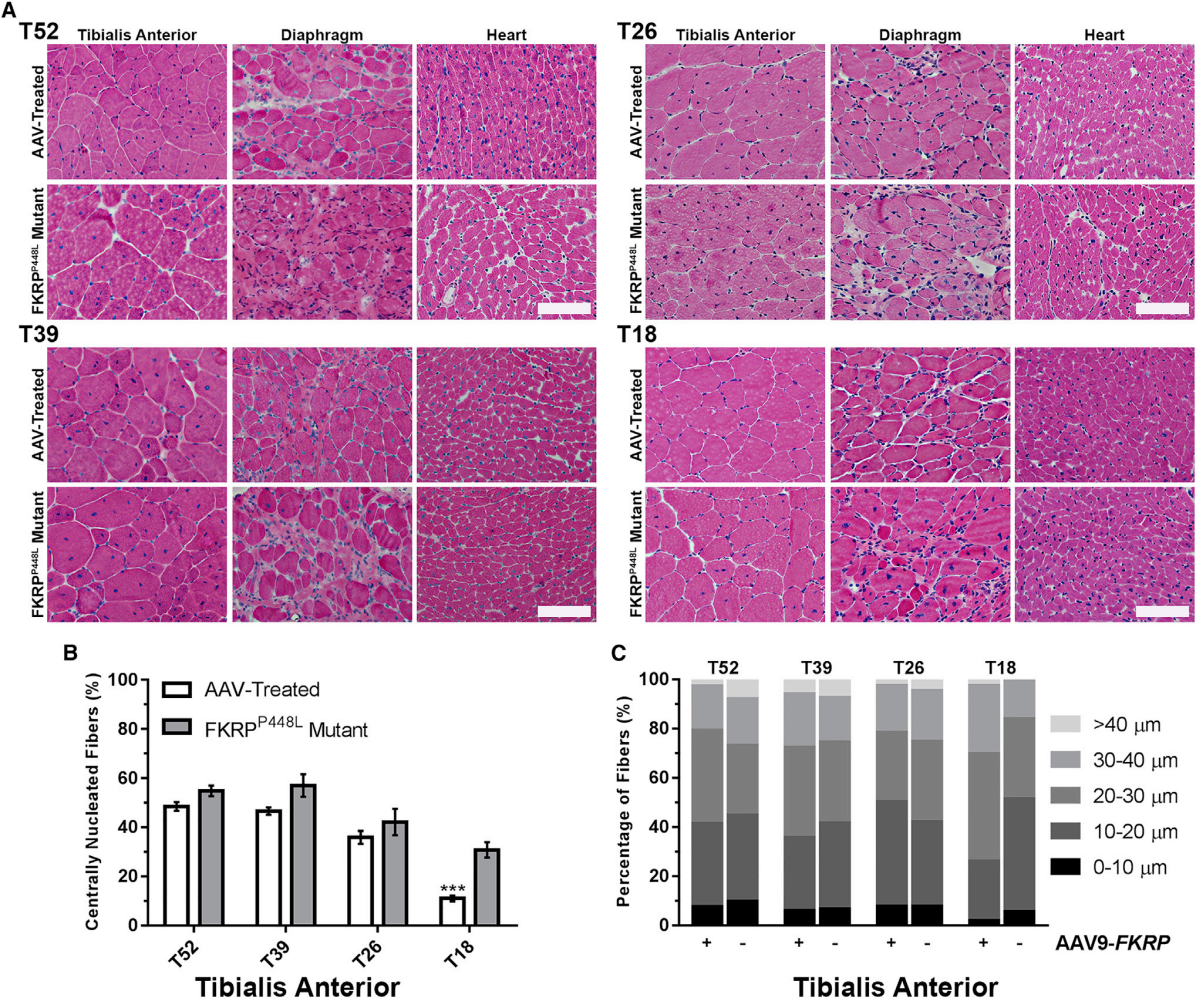
*FKRP* Gene Therapy Improves the Internal Structure and Hyper-/Hypotrophy of Skeletal and Cardiac Muscle Fibers (A) Cross-sections from tibialis anterior, diaphragm, and heart tissue of AAV-treated and FKRP^P448L^ mutant mice stained with H&E. Scale bars, 100 μm. (B) Quantification of centrally nucleated fibers in AAV-treated and untreated FKRP^P448L^ mutant tibialis anterior muscles (n = 4). ***p ≤ 0.001 (Student’s t test, each condition versus age-matched FKRP^P448L^ mutant mice). (C) The equivalent radius of individual myofibers in tibialis anterior muscles from FKRP^P448L^ mutant mice injected with AAV9-*FKRP* (+) or untreated (−) for each cohort (n = 4).

Similar to the clinical severity observed in patients with LGMD2I, FKRP^P448L^ mutant mice exhibit a mild-to-moderate phenotype of muscular dystrophy represented as myofiber regeneration/degeneration, significant fiber size variability, mononuclear cell infiltration, and pronounced fibrosis in the later stages of disease progression. Examination of the muscle morphology by H&E staining indicates that degeneration/regeneration in the limb skeletal muscles of the untreated FKRP^P448L^ mutant begins to plateau around 26 weeks of age, as demonstrated by the presence of large areas of necrotic fibers and a considerable number of centrally nucleated fibers (Figure 2A). An increase in fibrosis becomes clearly recognizable in the diaphragm and functional defects in skeletal and cardiorespiratory systems become detectable from the age of 26 weeks onward. Conversely, systemic *FKRP* gene delivery ameliorated pathological phenotypes of the dystrophic skeletal muscles of all mice from both early- and late-treated cohorts, which included a normalization of fiber size distribution, a reduction in centralized nuclei, and less pronounced fibrosis.

Quantitative analysis of the tibialis anterior muscles from each cohort of untreated FKRP^P448L^ mutant mice showed that the number of centrally nucleated fibers increased from younger to older cohorts, with 30.8% ± 3.1%, 42.2% ± 5.4%, 57.0% ± 4.6%, and 54.9% ± 2.1%, from T18 to T52, respectively, reaching a plateau around 39 weeks of age (Figure 2B). In contrast, the T18 cohort from the AAV-treated mice contained a significantly smaller percentage (11.0% ± 1.2%, p ≤ 0.001) of centrally nucleated fibers. This percentage also decreased in the T26 (36.0% ± 2.6%, p = 0.3399), T39 (46.6% ± 1.5%, p = 0.0730), and T52 (48.5% ± 1.8%, p = 0.0633) cohorts from the AAV-treated mice, but it lacked significance compared to the age-matched untreated controls. The similar percentage of centrally nucleated fibers in the two older (T39 and T52) cohorts is consistent with the notion that muscle degeneration and regeneration reaches a plateau around 26 weeks of age in FKRP^P448L^ mutant mice and that the time required for peripheralization of nuclei could take several months or longer to complete. Quantitative analysis of the collective myofiber radius revealed a decreased proportion of fibers with large and small diameters representing hypertrophy and regenerating fibers, respectively, in all cohorts of the AAV-treated muscles when compared to untreated FKRP^P448L^ mice (Figure 2C). The population of large fibers (>40 μm) was very small in the T18 cohort, and the difference between treated and untreated FKRP^P448L^ mutant mice was not clear. Together, these results suggest a normalization of fiber size distribution, representing a clear deceleration or halting of prolonged cycles of regeneration/degeneration.

**Figure 3:**
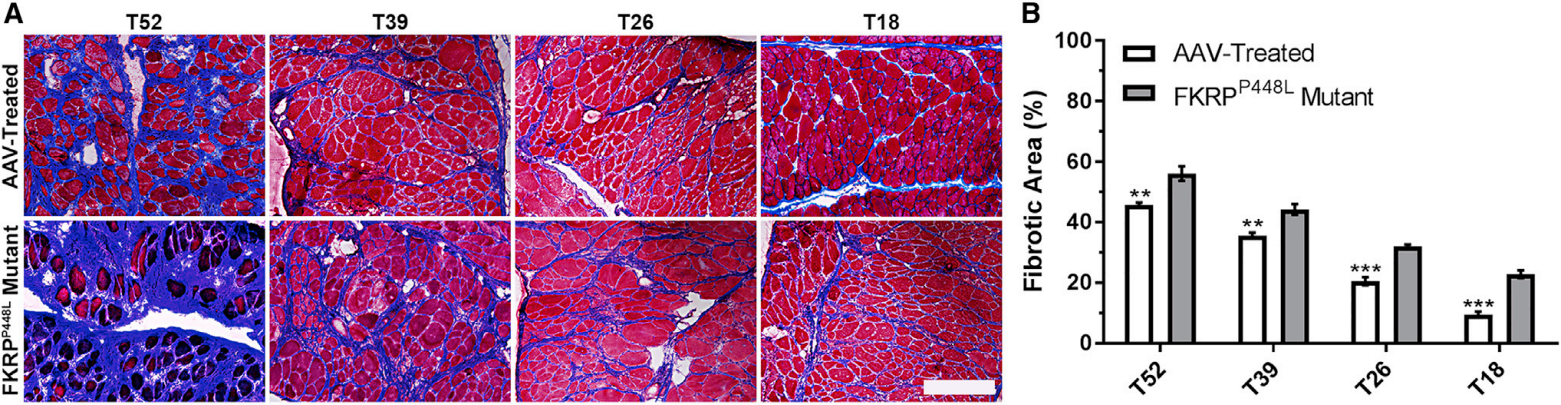
Gene Therapy with AAV9-*FKRP* Slows the Progression of Fibrosis in the Diaphragm (A) Masson’s trichrome staining of diaphragm tissue of AAV-treated and FKRP^P448L^ mutant mice in each cohort. Scale bar, 200 μm. (B) Quantification of the fibrotic area of the diaphragm in AAV-treated and FKRP^P448L^ mutant mice for each cohort (n = 4). **p ≤ 0.01; ***p ≤ 0.001 (Student’s t test, each condition versus age-matched FKRP^P448L^ mutant mice).

Another histological hallmark of the FKRP^P448L^ mutant mouse is pronounced and progressive fibrosis in the diaphragm as the mouse ages. To evaluate fibrotic changes after treatment, Masson’s trichrome staining was performed on tissue sections of the diaphragm from each cohort (Figure 3A). The results show a significant reduction in the amount of collagen present within AAV-treated cohorts compared with the untreated cohorts. Quantitative data confirmed that deposition of connective tissues follows a linear increase with age in both AAV-treated and untreated FKRP^P448L^ mutant mice (Figure 3B). More importantly, areas of connective tissue were significantly more prominent in untreated FKRP^P448L^ mutant mice compared to AAV-treated mice within each age cohort (T52: 56.2% ± 2.4% versus 45.7% ± 0.7%, p = 0.0060; T39: 44.2% ± 1.7% versus 35.5% ± 0.9%, p = 0.0051; T26: 32.0% ± 0.7% versus 20.5% ± 1.3%, p = 0.0002; and T18: 22.8% ± 1.3% versus 9.5% ± 0.9%, p = 0.0002). Quantitative comparisons additionally reveal that AAV-mediated delivery of *FKRP* to FKRP^P448L^ mutant mice reduces the level of fibrosis of each treatment cohort to a similar level of a younger untreated cohort corresponding to the treatment intervention time point (Table S1). Collectively, these results demonstrate that timely administration of an intervention that restores functionally glycosylated α-DG can slow or halt the development of fibrosis in the diaphragm before irreversible damage can occur.

### Gene Therapy with AAV9-*FKRP* Improves Muscle Function

**Figure 4:**
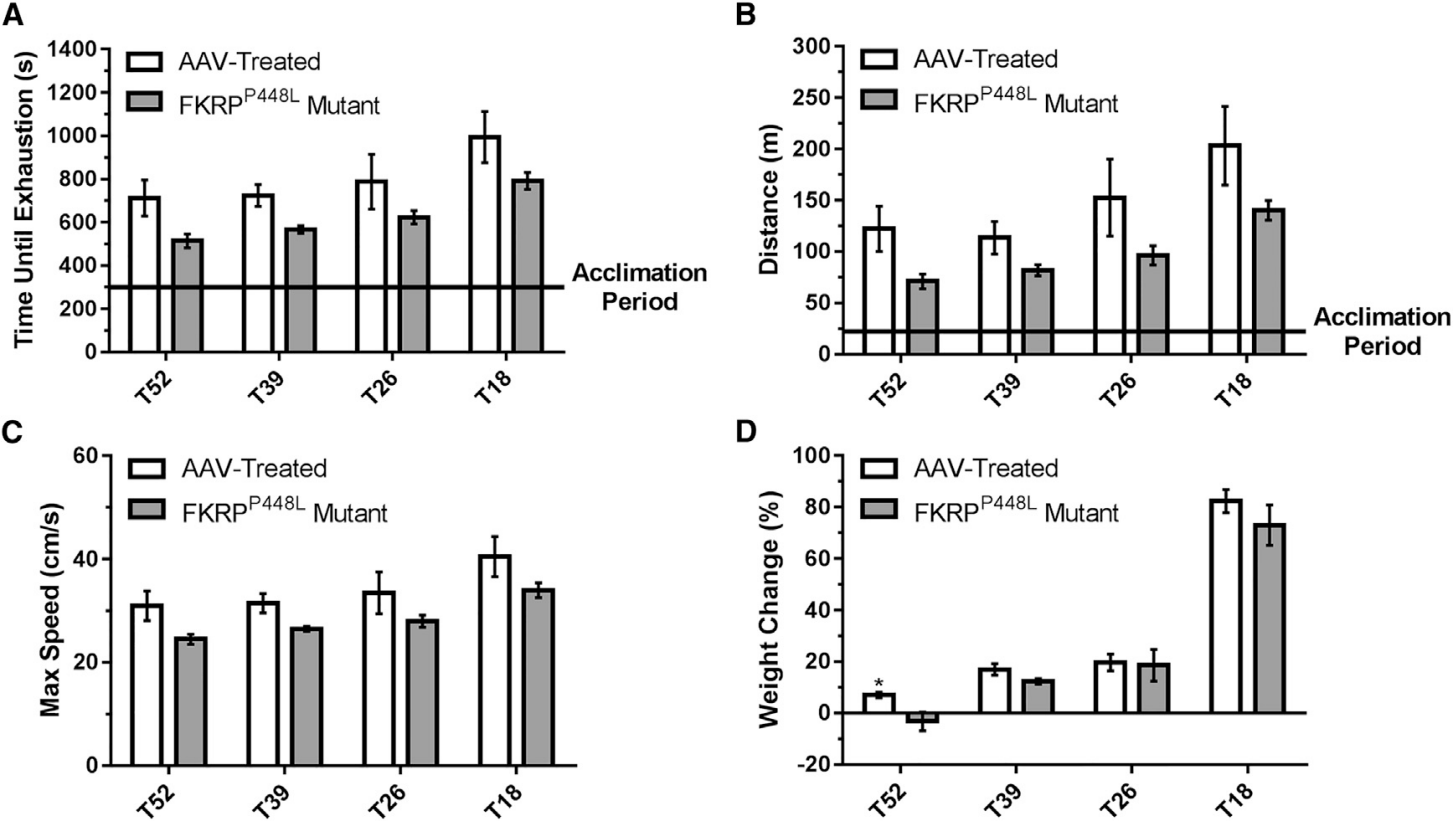
Improvement of Muscle Performance and Body Weight (A–C) Treadmill exhaustion test assessing the running time until exhaustion (A), distance covered (B), and maximum running speed (C) of AAV-treated and FKRP^P448L^ mutant mice in each cohort. (D) Percent change in body weight observed from initial treatment to euthanasia. *p ≤ 0.05 (Student’s t test, each condition versus age-matched FKRP^P448L^ mutant mice).

To assess the effect of rescuing functionally glycosylated α-DG on physical function, we conducted treadmill exhaustion tests 11 weeks post-injection. Initially, the mice were allowed to undergo an acclimation period, after which the test was started and mice were run to the point of exhaustion. The untreated FKRP^P448L^ mutant mice showed a reduced tolerance to exercise compared with AAV-treated mice (Figures 4A–4C). On average, mice from each AAV-treated cohort ran approximately 20%–28% longer at higher speeds than age-matched untreated FKRP^P448L^ mutant mice. Also, AAV-treated mice were able to run longer distances than age-matched untreated FKRP^P448L^ mutant mice, although a gradual decline was observed for both as age increased (untreated FKRP^P448L^ mutant versus AAV-treated mice, respectively: T18: 140.3 ± 9.6 versus 203.3 ± 38.4 m, p = 0.1623; T26: 96.3 ± 9.5 versus 152.5 ± 37.5 m, p = 0.1962; T39: 81.8 ± 5.5 versus 113.5 ± 15.9 m, p = 0.1075; and T52: 71.0 ± 7.2 versus 122.3 ± 22.0 m, p = 0.0690). Together, these data indicate that muscle impairment can be ameliorated at all stages of disease progression.

The assessment of body weight (measured every 6–7 weeks) was reported as a percent change relative to baseline and revealed that all study groups gained body mass over the 13-week observation period, except for untreated FKRP^P448L^ mutant mice in the T52 cohort (Figures 4D and S2). No significant difference was observed between the AAV-treated and untreated FKRP^P448L^ mutant mice in the T18, T26, or T39 cohorts.

### Effect of AAV9-*FKRP* Treatment on Respiratory and Cardiac Parameters

To evaluate respiratory function, unrestrained and conscious AAV-treated and untreated FKRP^P448L^ mutant mice were monitored by whole-body plethysmography. This non-invasive technique provides a comprehensive pulmonary analysis by measuring sensitive respiratory parameters that include breathing frequency (F; the number of breaths per minute), tidal volume (TV; amount of air inhaled and exhaled normally at rest), minute volume (MV; amount of air inhaled and exhaled per minute), peak inspiratory flow (PIF; maximal flow rate achieved during the inspiratory cycle), and peak expiratory flow (PEF; maximal flow rate achieved during the maximally forced expiration initiated at full inspiration) (Table 1). Analysis of the results indicates an improvement trend in a majority of the respiratory parameters of the early-stage AAV-treated cohorts (T18 and T26) when compared to age-matched untreated FKRP^P448L^ mutant mice. However, no statistical significance was reached with the small size of the cohorts. Consistent with more progressive fibrosis in the diaphragm as observed from H&E and Masson’s trichrome staining, minimal or no improvement at all is observed with the late-stage AAV-treated cohorts (T39 and T52). Assessment of cardiac morphology and function was conducted via echocardiography and summarized (Table S2). The results indicate that there was no significant difference in cardiac morphology or function between the AAV-treated and untreated FKRP^P448L^ mutant mice cohorts at each time point.

**Table 1:** Assessment of Respiratory Function

Post-injection Time Point^a^	T18		T26		T39		T52	
	AAV Treated	FKRP^P448L^ Mutant	AAV Treated	FKRP^P448L^ Mutant	AAV Treated	FKRP^P448L^ Mutant	AAV Treated	FKRP^P448L^ Mutant
**F (Breaths/min)**								

1 week	248.56 ± 5.86	253.70 ± 3.36	258.55 ± 5.44	260.36 ± 6.12	279.01 ± 13.32	271.63 ± 5.22	276.15 ± 5.93	270.64 ± 8.90
13 weeks	273.73 ± 6.08	275.58 ± 8.15	244.77 ± 1.15	268.89 ± 9.32	284.20 ± 18.54	289.85 ± 10.91	262.74 ± 12.80	276.03 ± 22.34
Percent change	10.1	8.6	−5.3	3.3	1.9	6.7	−4.9	2.0

**TV (mL)**								

1 week	0.16 ± 0.01	0.16 ± 0.01	0.17 ± 0.01	0.18 ± 0.00	0.25 ± 0.02	0.24 ± 0.02	0.22 ± 0.02	0.21 ± 0.02
13 weeks	0.26 ± 0.02	0.23 ± 0.01	0.27 ± 0.03	0.25 ± 0.01	0.26 ± 0.03	0.24 ± 0.01	0.21 ± 0.01	0.21 ± 0.02
Percent change	60.1	45.6	59.1	34.1	1.7	−0.2	−5.4	2.0

**MV (mL)**								

1 week	40.29 ± 2.30	39.78 ± 1.67	43.13 ± 1.80	47.98 ± 1.66	71.15 ± 8.53	64.26 ± 6.34	61.92 ± 5.74	57.05 ± 6.33
13 weeks	71.26 ± 6.96	62.73 ± 5.06	64.76 ± 6.55	67.01 ± 3.29	74.10 ± 10.75	68.23 ± 5.39	55.52 ± 4.86	60.45 ± 10.00
Percent change	76.9	57.7	50.1	39.7	4.1	6.2	−10.3	5.9

**PIF (mL/s)**								

1 week	5.98 ± 0.19	5.95 ± 0.23	6.34 ± 0.18	6.78 ± 0.35	6.87 ± 0.34	6.75 ± 0.45	5.78 ± 0.45	5.68 ± 0.51
13 weeks	6.73 ± 0.53	6.32 ± 0.25	7.08 ± 0.61	6.38 ± 0.37	6.40 ± 0.43	5.57 ± 0.18	5.10 ± 0.15	5.00 ± 0.35
Percent change	12.7	6.4	11.7	−6.0	−6.8	−17.5	−11.8	−12.0

**PEF (mL/s)**								

1 week	3.38 ± 0.18	3.92 ± 0.14	3.94 ± 0.23	4.13 ± 0.25	4.01 ± 0.32	4.08 ± 0.31	3.57 ± 0.23	3.42 ± 0.18
13 weeks	4.12 ± 0.32	4.07 ± 0.19	4.05 ± 0.30	3.81 ± 0.20	4.08 ± 0.37	4.00 ± 0.13	3.35 ± 0.17	3.61 ± 0.21
Percent change	21.9	3.9	2.6	−7.9	1.6	−2.1	−6.2	5.5

Respiratory function parameters in AAV-treated and FKRP^P448L^ mutant mice for T18, T26, T39, and T52 cohorts at post-injection time point intervals of 1 and 13 weeks. F, breathing frequency; MV, minute volume; PEF, peak expiratory flow; PIF, peak inspiratory flow; TV, tidal volume.

a Percent change from 1 to 13 weeks.

## Discussion

Great advancements have been made in transforming gene replacement therapy into an efficient, sustainable method for treating dystrophic disorders. However, many questions remain to be answered before the therapeutic approach can be effective in a clinical study focused on a majority of patients with muscular dystrophy. Pre-clinical studies related to muscular dystrophy have proven that AAV-mediated delivery can result in transgenic expression at therapeutic levels in body-wide skeletal muscles and organs of interest. Recently, our research group and collaborators reported that AAV-mediated delivery of *FKRP* was able to effectively express sufficient levels of FKRP protein, triggering the restoration of functional glycosylation of α-DG in mouse models of LGMD2I containing point mutations of proline to leucine at position 448 (FKRP^P448L^) and leucine to isoleucine at position 276 (FKRP^L276I^).^22^, ^23^ Administration at an early stage of the disease ameliorated disease progression, with significant improvement in dystrophic pathology, serum creatine kinase levels, and muscle function void of any deleterious evidence. The results showed that transgenic FKRP expression, levels of functionally glycosylated α-DG, and other therapeutic effects persisted long after treatment. However, disease progression of muscular dystrophy is the result of muscle wasting and accumulation of fibrotic tissue, both of which are likely to have a profound effect on the efficacy of gene therapy. Consequently, knowing the degree of efficacy that can be achieved in individuals with different severities or at different stages of disease progression is critical.

To our knowledge, we are the first to demonstrate the phenotypic correction of *FKRP*-related muscular dystrophies at multiple stages of disease progression in a mouse model by a single intravenous administration of an AAV vector. The results of this study address the important issue as to whether gene replacement will have similar efficacies at different stages of disease progression. This is especially important because *FKRP*-related muscular dystrophies exhibit a wide spectrum of clinical severity, variability in age at onset, and varying degrees of myogenic atrophy. These variables significantly complicate the clinical study process, including population selection, dose determination, and comprehensive evaluation of the results. However, these challenges can be overcome by understanding the effect of the underlying variables to the therapy in animal models relevant to clinical manifestations. We propose that several aspects of the disease can be conjectured to affect the efficiency of transgene expression and efficacy of the therapy. Specifically, pathological changes with aging and an increasing amount of fibrosis with disease progression are two important factors that can determine the efficiency of transgene expression and its functional impact on diseased muscles. Our FKRP^P448L^ mutant mouse model serves this purpose well by presenting dystrophic phenotypes that closely resemble those demonstrated in patients with LGMD2I. This model exhibits severely reduced expression of functionally glycosylated α-DG in all skeletal and cardiac muscles, along with progressive degeneration as the mouse ages, which is associated with a gradual loss of muscle mass and an increase in fibrosis, especially in the diaphragm.

Our results indicate that administration of AAV9-*FKRP* at a dose of 2.5 × 10^13^ vg/kg has the greatest therapeutic effect in the youngest T18 cohort. This includes the highest levels of restoration of functional glycosylation of α-DG, maximum improvement in both skeletal and respiratory functions, and prevention of dystrophic pathology. Treatment at later stages of disease progression also improves pathology and functions, but at reduced rates. More importantly, the therapeutic effect could only reach a level similar to the point at which the treatment was initiated. For example, AAV-treated mice in the T39 cohort (treated at 26 weeks of age) showed a reduced percentage of centrally nucleated fibers as well as reduced areas of fibrotic tissue, but both percentages remained higher than those of the untreated FKRP^P448L^ mutant T26 cohort. This could be due, in part, to the fact that even though AAV9 demonstrates early expression (within 7 days post-injection), maximum vector expression may not occur until 100 days post-injection.^28^ A similar trend is evidenced in the functional indices assessed by the treadmill exhaustion test. Therefore, the results support the notion that it may be difficult for an AAV gene therapy to reverse existing pathological changes such as fibrosis and fragmented fibers that can hinder the function of diseased muscles. It remains to be investigated as to whether a higher dose and prolonged period of treatment could further improve functional outcomes of skeletal, respiratory, and cardiac muscles, reaching statistical significance in all cohorts.

In general, the correction of dystrophic muscle pathology is highly dependent on the restoration of functional glycosylation of α-DG. We observed that the rescue of functional glycosylation of α-DG was variable between tissues in the treated mice and the amount was highly dependent on the age at which the mouse was treated. The reason behind the lower levels of functionally glycosylated α-DG in muscles, especially skeletal muscles, of the late-stage treated cohorts is not clearly understood. One possible explanation is that there is a lower level of infectivity of AAV in the muscles of older mice, which have experienced progressive accumulation of the extracellular matrix, thereby reducing the accessibility of the virus to dystrophic fibers. Another possible explanation is that the persistent degeneration is expected to lead to a loss of viral vectors, especially before transgene expression is able to achieve its protective effect. This theory is consistent in tissues where degeneration and fibrosis are minimal and progress slowly, such as in the case of the cardiac muscles, which showed relatively similar levels of functionally glycosylated α-DG in all AAV-treated cohorts. However, such hypotheses cannot be fully validated until we are able to understand the amount of virus and the localization of viral vectors within each individual muscle. The effort to quantify the total number of viral vector copy numbers has been met with difficulty for interpretation, as localization of the virus, whether in connective tissue cells or muscle fibers, could not be determined by whole-tissue analysis. The expression levels of the FKRP transgene are currently the only data we have, which appear to show similar levels in the same muscle tissue of all AAV-treated cohorts. Therefore, muscle-specific and age-related variation in the efficiency of glycosylation of α-DG cannot be excluded. However, it should be noted that the levels of FKRP protein in the tibialis anterior muscles are very low and only detectable by western blot analysis utilizing an FKRP (C-terminal) antibody. Perhaps also relevant is that the FKRP protein is detected as a dimer, judged by the molecular weight, as opposed to the monomer form of FKRP, which is undetectable. It is possible that the detectable dimer form of FKRP might not represent the actual amount or even active form of FKRP expressed by the AAV vector.

Despite the difficulty in determining the mechanism(s) involved, there is a clear indication that levels of functional glycosylation of α-DG can be considerably lower in severely affected muscles and at later stages of disease progression. This has significant clinical implications, considering the fact that most patients, even those with relatively mild LGMD2I, are likely to have more severe muscle degeneration and extensive extracellular matrix accumulation compared to the muscles of a dystrophic mouse model, in which significant fibrosis is largely limited to the diaphragm. The efficiency of AAV viral infection, transgene expression, and/or glycosylation of α-DG in patients is likely to be considerably lower and even more variable than that observed in the skeletal muscles of the late-stage treated cohorts. Our results strongly support the notion that early treatment is critical for achieving high efficacy. Encouragingly, AAV gene therapy at all stages of disease progression has the ability to maintain existing muscle mass and improve, albeit limited, muscle function as shown in our study. One possible alternative to compensate for the diminishing efficiency in transgene expression and glycosylation of α-DG in advanced diseases could be the use of a higher viral dosage, which brings with it more risks that include non-target tissue expression, loss of efficacy, and the potential to trigger anti-capsid cytotoxic T lymphocyte responses.^29^ Studies related to this issue are currently being investigated by our research group.

The effective consequence of gene replacement therapy is the expression of a therapeutic transgene product, which is the FKRP protein in our study. Normally, this product would represent an essential biomarker for the assessment of efficacy in a clinical trial. Detection of the FKRP transgene by both immunohistochemistry and western blot has been fairly straightforward using a strong promoter such as cytomegalovirus (CMV), which we have previously reported.^22^ However, for clinical applications, a muscle-specific promoter such as the CK7-based cassette is highly preferred for selective expression in targeted dystrophic muscles.^30^ Unfortunately, the CK7-based cassette is slightly more active but very weak compared with the CMV cassette. At the dose of 2.5 × 10^13^ vg/kg used in this study, FKRP transgene expression can only be detected weakly by western blot and cannot be convincingly identified in almost all muscle fibers by immunohistochemistry. In contrast, detectable levels of functional glycosylation of α-DG are demonstrated without much difficulty. Previous immunohistochemical colocalization results have shown that restoration of functional glycosylation of α-DG can be achieved in fibers without detectable signals for FKRP.^22^ This suggests that detectable levels of FKRP protein are not necessarily required for the restoration of normal levels of functional glycosylated α-DG and subsequent rescue of the dystrophic phenotype. It should be noted that this does not contradict the observed efficacy shown by the restored functional glycosylation of α-DG and improvement in muscle function. In fact, one major obstacle for *FKRP*-related muscular dystrophies has been the inability to detect endogenous FKRP protein despite great efforts.^31^ In the last decade, many antibodies against FKRP peptide sequences have been produced, including FKRP829-STEM (Dr. Derek J. Blake, Cardiff University), FKRP (C-16) (Santa Cruz Biotechnology), and FKRP (C-terminal) antibody (our laboratory), none of which have convincingly detected endogenous FKRP. This suggests that the level of functionally glycosylated α-DG may be a more reliable and direct biomarker for therapeutic efficacy until a more sensitive and consistent detection method for FKRP can be developed.

In conclusion, this study provides direct evidence that a single-dose therapy can result in viable FKRP expression that is able to successfully generate functional glycosylation of α-DG and ameliorate the dystrophic phenotypes at multiple stages of disease progression. Our results are particularly important with regard to the clinical translation of *FKRP* gene therapy, suggesting that early therapeutic interventions are critical for achieving high efficacy and are likely to have the best chance to counteract the deleterious effects of LGMD2I. Conversely, substantial correction of the disease pathology is unlikely to occur in the advanced stages of disease progression, where muscle tissue is progressively replaced by adipose and fibrotic tissue. Nevertheless, limited benefits are achievable for more advanced stages of the disease.

## Materials and Methods

### Study Design

#### Rationale and Design of Study

This was a proof-of-concept study designed to search for possible differences among experimental treatment groups. Animals were assigned to treatment groups on the basis of availability of gene replacement vector.

#### Randomization and Blinding

This was an open-label and non-randomized study.

#### Replication

Repeated functional measures were conducted over time in animals as indicated. Technical replicates were performed in immunohistochemical, western blot, quantitative real-time PCR, and histological analyses.

#### Ethics Statement

All animal studies were approved by the Institutional Animal Care and Use Committee (IACUC) of Carolinas Medical Center. All mice were housed in the vivarium of Carolinas Medical Center according to animal care guidelines of the institute. Food and water were available ad libitum during all phases of the study.

### Mouse Model

FKRP^P448L^ mutant mice were generated by the McColl-Lockwood Laboratory for Muscular Dystrophy Research.^25^, ^26^ These mice contain a homozygous missense mutation (c.1343C > T, p.Pro448Leu) in the *FKRP* gene with the floxed neomycin resistant (Neo^r^) cassette removed from the insertion site.

### Construction of AAV Vector and Administration

The recombinant AAV vector construction was performed as described previously.^32^ Full-length human *FKRP* cDNA was synthesized for high expression in mice and was subsequently subcloned into a single-stranded AAV9 vector under control of a muscle-specific CK7 promoter. Recombinant AAV vector stocks were produced according to the three-plasmid cotransfection method reported previously.^33^ The viral particles were purified twice by cesium chloride density gradient ultracentrifugation using the previously published protocol.^34^ Vector titers were determined by both dot-blot and real-time PCR methods using previously published protocols.^35^, ^36^ The concentration of viral vectors was kept in the range of 5 × 10^12^ vg/mL and stored at −80°C until future use.

FKRP^P448L^ mutant mice were administered with AAV9-*FKRP* at a dose of 2.5 × 10^13^ vg/kg via a single tail-vein injection at four different age points: 5, 13, 26, and 39 weeks (n = 4 for each cohort; 2 male and 2 female). Mice were euthanized 13 weeks after injection. All untreated FKRP^P448L^ mutant mice (n = 4 for each cohort; 2 male and 2 female) were euthanized at the same age point as the AAV-treated mice. FKRP^P448L^ mutant mice (n = 4 for each cohort; 2 male and 2 female) aged 5 and 13 weeks were used as controls for fibrotic area comparisons. C57BL/6 mice (n = 2; 1 male and 1 female) aged 14 weeks were used as controls for western blot normalization.

### Immunohistochemical and Western Blot Analysis

Tissues were dissected and snap-frozen in dry-ice-chilled 2-methylbutane. Tissues were cryosectioned (6-μm thick), positioned on glass microscope slides, and then stored at −80°C until future use. Antibodies used in this study were obtained as follows: mouse monoclonal α-DG (clone IIH6C4) was from EMD Millipore, rabbit polyclonal actin and laminin was from Sigma-Aldrich, rabbit polyclonal glyceraldehyde 3-phosphate dehydrogenase (GAPDH) was from Thermo Fisher Scientific, and an affinity-purified rabbit polyclonal FKRP (C-terminal, sequence: NPEYPNPALLSLTGG) was produced in our laboratory.

For immunohistochemical detection, frozen tissue sections were incubated with 1× Tris-buffered saline (TBS) for 5 min and then immediately blocked with 10% normal donkey serum diluted in 1× TBS for 30 min. Sections were then incubated overnight at 4°C with primary antibodies IIH6C4 (1:200) or laminin (1:200) diluted in 1× TBS. Sections were washed two times with 1× TBS and appropriate secondary antibodies were incubated at room temperature for 1 hr. Sections were washed three times with 1× TBS and mounted with fluorescence mounting medium (Dako) containing 1× DAPI. Images were visualized using an Olympus BX51/BX52 fluorescence microscope (Opelco) and were captured using the Olympus DP70 digital camera system (Opelco).

For western blot analysis, tissues were rapidly homogenized in extraction buffer (50 mM Tris-HCl, pH 8.0, 150 mM NaCl, 1% SDS, and 1% Triton X-100) supplemented with 1× protease inhibitor cocktail (Sigma-Aldrich). Non-dissolved protein was removed by centrifugation (22,000 × *g* for 15 min at 4°C). Protein concentrations were measured using a NanoDrop 2000 spectrophotometer (Thermo Fisher Scientific). For each lane, approximately 30 μg protein was loaded, separated on a 4%–20% Tris-glycine polyacrylamide gel (Life Technologies), and immunoblotted. Nitrocellulose membranes were blocked with Protein-Free T20 (TBS) blocking buffer (Thermo Fisher Scientific) or 3% milk in 1× TBS with Tween 20 (0.05%) for 1 hr at room temperature and then incubated with the following primary antibodies overnight at 4°C: IIH6C4 (1:1,000), FKRP (C-terminal) (1:400), actin (1:8,000), and GAPDH (1:2,000). Appropriate horseradish peroxidase (HRP)-conjugated secondary antibodies were applied to the membranes for 1 hr. All blots were developed by electrochemiluminescence immunodetection (PerkinElmer), exposed to BioMax Light Film (Sigma-Aldrich), and processed by a Mini-Medical imaging system (AFP Imaging) or by manual film processing. The detection of actin and GAPDH confirmed that a similar amount of protein was loaded for each sample.

### Quantitative Reverse Transcriptase PCR Assays

Total RNA was extracted from tibialis anterior tissues using TRIzol reagent (Life Technologies), and cDNA was synthesized using a High-Capacity RNA-to-cDNA kit (Applied Biosystems). PCR analysis was performed using PowerUp SYBR Green Master Mix (Life Technologies), and amplified PCR products were quantified and normalized using GAPDH as a control. Cycling conditions for FKRP transgene expression were as follows: initial uracil-DNA glycosylase (UDG) activation (50°C/2 min), Dual-Lock DNA polymerase (95°C/2 min), 40 cycles (95°C/15 s; 55°C/15 s; and 72°C/1 min). This was followed by melting curve analysis starting at 65°C and increasing to 95°C at a programmed rate of 0.1°C/s. The primer pair used is 5′-CCACAGCACAGACAGACACT-3′ (forward) and 5′-CTCCGGGGCATCCTTAGAAA-3′ (reverse) (Integrated DNA Technologies). All reactions were performed in triplicate.

### Histopathological and Morphometric Analysis

Frozen tissue sections were processed for H&E staining using standard procedures. For H&E images, one representative ×40 magnification image from each cohort was used. For morphometric analysis, tibialis anterior muscles were subject to immunostaining as described above with rabbit anti-laminin primary antibody (Sigma-Aldrich) and an anti-rabbit IgG (H+L) secondary antibody, Alexa Fluor 594 conjugate (Life Technologies). Muscle cross-sectional fiber radii and the percentage of myofibers with centrally located nuclei were quantified manually from photographs taken at ×10 magnification (average of ≥450 fibers). Images were visualized using an Olympus BX51/BX52 fluorescence microscope and were captured using the Olympus DP70 digital camera system. All measurements and calculations were conducted using MetaMorph Microscopy Automation and Image Analysis Software (version 7.7.0.0; Molecular Devices).

### Masson’s Trichrome Staining

Tissue slides were thawed and fixed in 10% neutral buffered formalin overnight at room temperature, rinsed with water, and then baked at 56°C in Bouin’s fixative for 1 hr. Slides were rinsed with water and then dipped in Weigert’s iron hematoxylin for 10 min. Slides were washed twice in deionized water and then incubated in Biebrich scarlet-acid fuchsin solution for 30 min. After a brief washing in deionized water, slides were put in phosphotungstic phosphomolybdic acid for 12 min and stained with aniline blue for 20 min. Slides were washed in deionized water and subsequently placed in acidified ethanol containing 1% glacial acetic acid for 2 min. Slides were then dipped three times each in 95% ethanol, 100% ethanol, and xylene (Sigma-Aldrich). All reagents were acquired from Poly Scientific R&D unless otherwise stated. Masson’s trichrome was used to determine the amount of fibrosis within the diaphragm, which was calculated from the entire muscle cross-sectional area and quantified using ImageJ software (Java version 1.6.0_20; NIH). The color threshold of the images was adjusted in ImageJ using the hue/saturation/brightness filter sets dividing the image into two-color ranges. Particles on the created masks were subsequently analyzed and the total areas were used to create the blue-to-total ratio indicating the percentage of fibrous tissue. One representative ×20 magnification image for each diaphragm per animal was used.

### Treadmill Exhaustion Test

All mice were subjected to a treadmill exhaustion test. Mice were placed on the belt of a five-lane motorized treadmill (Columbus Instruments) supplied with shock grids mounted at the back of the treadmill, which delivered a 0.2-mA current to provide motivation for exercise. Initially, the mice were subjected to an acclimation period (time, 5 min; speed, 8 cm/s; distance, ∼24 m; and incline, 0°). Immediately after the acclimation period, the test commenced at a speed of 8 cm/s and was subsequently increased 2 cm/s every minute until exhaustion. The test was stopped and the time to exhaustion was determined when the mouse remained on the shock grid at the back of the treadmill for 5 s without attempting to re-engage the treadmill or if the mouse repeatedly failed to make it at least halfway up the treadmill lane. Stop times were rounded to the nearest 15-s interval.

### Whole-Body Plethysmography

Respiratory functional analysis in conscious, freely moving mice was measured using a whole-body plethysmography technique. The plethysmograph apparatus (emka Technologies) was connected to a ventilation pump for the purpose of maintaining constant air flow, a differential pressure transducer, a USBamp signal amplifier, and a personal computer running iox2 software (emka Technologies) with the respiratory flow analyzer module, which was used to detect pressure changes due to breathing and recording the transducer signal. 20 mL of air was injected and withdrawn via a 20-mL syringe into the chamber for the purpose of calibration. Mice were placed inside the plethysmograph chamber and allowed to acclimate for 5 min in order to minimize any effects of stress-related changes in ventilation. Resting ventilation was measured for a duration of 15 min after the acclimation period. Body temperatures of all mice were assumed to be 37°C and to remain constant during the ventilation protocol.

### Transthoracic Echocardiography

Transthoracic echocardiography was performed on anesthetized (1%–2% isoflurane) mice using the SonixTablet Ultrasound System (BK Ultrasound). Mice were held in a supine position on a mouse monitor pad, the anterior chest wall was shaved, warm ultrasound gel was applied to the chest area, and the transducer probe was placed over the left hemithorax. Multiple short axis M-mode images of the left ventricle were obtained and these images were analyzed for left ventricular function parameters in triplicate. The heart was also imaged under B-mode with the B-mode placement imaging the heart at the level of the aortic sphincter. Cardiac examination time was approximately 15 min.

### Statistical Analysis

All data are expressed as means ± SEM unless stated otherwise. Statistical analyses were performed with GraphPad Prism software (version 7.01 for Windows; GraphPad Software). Individual means were compared using multiple Student’s t tests. Differences were considered to be statistically significant at p ≤ 0.05, p ≤ 0.01, or p ≤ 0.001.

## Author Contributions

Conceptualization, C.H.V. and Q.L.L.; Methodology, C.H.V. and Q.L.L.; Validation, C.H.V.; Formal Analysis, C.H.V. and W.X.; Investigation, C.H.V., W.X., and P.L.; Resources, Q.L.L. and X.X.; Data Curation, C.H.V. and W.X.; Writing – Original Draft, C.H.V.; Writing – Review & Editing, C.H.V. and Q.L.L.; Visualization, C.H.V.; Supervision, Q.L.L.; Project Administration, C.H.V. and Q.L.L.; Funding Acquisition, X.X. and Q.L.L.

## Conflicts of Interest

The authors declare no conflicts of interest.

## Supplemental Information

Document S1. Figures S1 and S2 and Tables S1 and S2

Document S2. Article plus Supplemental Information
