# Supplementary material for: Efficacy of Gene Therapy Is Dependent on Disease Progression in Dystrophic Mice with Mutations in the FKRP Gene
Source: Mol Ther Methods Clin Dev. 2017 Mar 8;5:31–42. doi: 10.1016/j.omtm.2017.02.002 (PMC5415313; doi:10.1016/j.omtm.2017.02.002)
Supplement: Document S1. Figures S1 and S2 and Tables S1 and S2 [file mmc1.pdf]

OMTM, Volume *5*

Supplemental Information

Efficacy of Gene Therapy Is Dependent on Disease Progression in Dystrophic Mice with Mutations in the FKRP Gene

Charles Harvey Vannoy, Will Xiao, Peijuan Lu, Xiao Xiao, and Qi Long Lu

SUPPLEMENTARY INFORMATION

Table of Contents

Figure S1. AAV9-*FKRP* vector construct 2

Table S1. Quantification of the fibrotic area in the diaphragm 3

Figure S2. Weight profiles 4

Table S2. Echocardiographic measurements 5

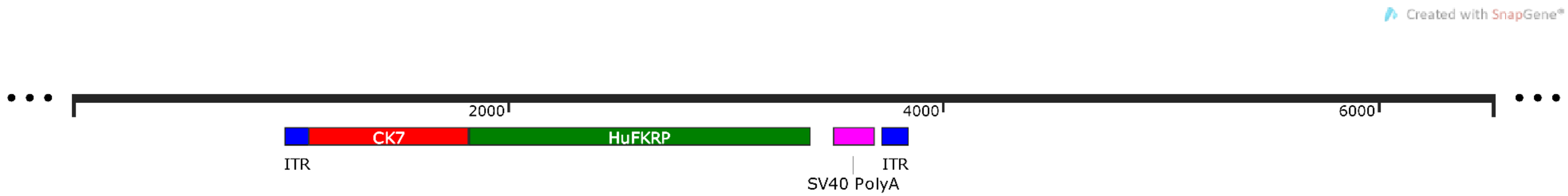

Figure S1. AAV9-*FKRP* vector construct. ITR, inverted terminal repeat of adenovirus; CK7, creatine kinase-based promoter; HuFKRP, full-length human *FKRP* coding sequence; SV40, Simian virus 40. Created with SnapGene®.

Age of Mice (Treatment Age)	52 Weeks (39 Weeks)	39 Weeks (n/a)	39 Weeks (26 Weeks)	26 Weeks (n/a)	26 Weeks (13 Weeks)	13 Weeks (n/a)	18 Weeks (5 Weeks)	5 Weeks (n/a)
AAV9-*FKRP*	+	-	+	-	+	-	+	-
Fibrotic Area (%)	45.7±0.7	44.2±1.7	35.5±1.0	32.0±0.7	20.5±1.3	19.9±0.9	9.5±0.9	11.1±1.5

Table S1. Quantification of the fibrotic area in the diaphragm of $FKRP^{P448L}$ mutant mice injected with AAV9-*FKRP* (+) or untreated (-) for each cohort (n = 4).

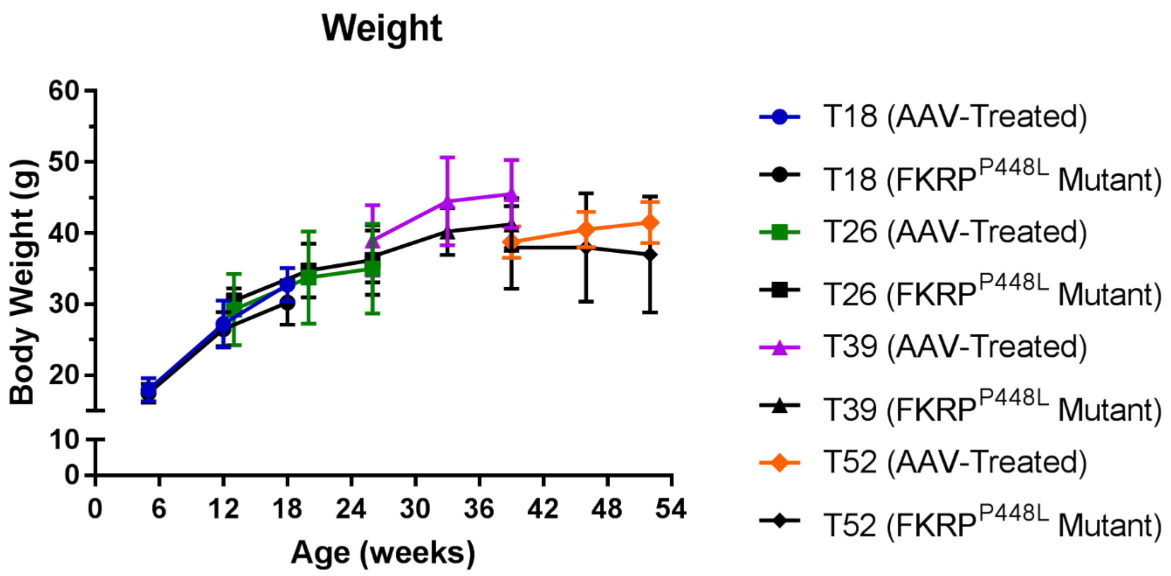

Figure S2. Weight profiles of the AAV-treated and $FKRP^{P448L}$ mutant mice in each cohort.

Cardiac Parameters	Post-Injection Time Point	T18		T26		T39		T52	
		AAV Treated	FKRP^{P448L} Mutant	AAV Treated	FKRP^{P448L} Mutant	AAV Treated	FKRP^{P448L} Mutant	AAV Treated	FKRP^{P448L} Mutant
HR (beats/min)	0 Week	508±20	528±8	513±4	519±12	497±15	526±6	536±6	522±11
	12 Week	510±13	495±5	452±21	509±15	537±15	510±12	520±13	491±21
	% Change	0.4%	-6.3%	-11.8%	-1.8%	7.9%	-3.0%	-3.0%	-5.9%
LVEF (%)	0 Week	64.5±2.8	65.3±3.3	64.1±1.0	58.6±3.1	60.7±3.1	61.7±3.9	59.5±1.9	62.4±4.1
	12 Week	58.0±2.9	56.2±2.5	52.1±3.5	57.1±1.8	57.1±2.1	61.8±2.9	50.7±1.6	55.8±2.8
	% Change	-10.1%	-13.9%	-18.8%	-2.5%	-5.9%	0.1%	-14.8%	-10.6%
SV (mL)	0 Week	0.016±0.002	0.016±0.001	0.024±0.004	0.017±0.002	0.019±0.000	0.020±0.002	0.018±0.003	0.023±0.003
	12 Week	0.017±0.001	0.019±0.002	0.013±0.001	0.021±0.001	0.023±0.002	0.018±0.001	0.021±0.002	0.023±0.003
	% Change	8.8%	16.2%	-43.9%	23.2%	21.6%	-9.2%	16.5%	-0.6%
CO (mL/min)	0 Week	8.07±1.18	8.50±0.68	12.06±2.11	8.94±1.09	9.56±0.28	10.34±1.01	9.42±1.36	11.77±1.40
	12 Week	8.74±0.56	9.23±1.22	5.88±0.23	10.68±0.44	12.55±1.22	9.08±0.47	10.56±0.81	11.18±1.74
	% Change	8.3%	8.6%	-51.3%	19.5%	31.3%	-12.2%	12.0%	-5.0%

Table S2. Echocardiographic measurements. HR, heart rate; LVEF, left ventricle ejection fraction; SV, stroke volume; CO, cardiac output.
